# Virtual clinical trials identify effective combination therapies in ovarian cancer

**DOI:** 10.1038/s41598-019-55068-z

**Published:** 2019-12-10

**Authors:** Emilia Kozłowska, Tuulia Vallius, Johanna Hynninen, Sakari Hietanen, Anniina Färkkilä, Sampsa Hautaniemi

**Affiliations:** 1 Research Program in Systems Oncology, Research Programs Unit, Faculty of Medicine, University of Helsinki, Helsinki, Finland; 2 Institute of Automatic Control, Silesian University of Technology, Akademicka 16, 44-100 Gliwice, Poland; 3 Department of Oncology and Radiotherapy, Turku University Hospital, University of Turku, Turku, Finland; 4 Department of Obstetrics and Gynecology, Turku University Hospital, University of Turku, Turku, Finland; 5 Department of Radiation Oncology, Dana-Farber Cancer Institute, Harvard Medical School, Boston, USA; 6 Department of Obstetrics and Gynecology, Helsinki University Hospital, Helsinki, Finland

**Keywords:** Cancer therapy, Computational models

## Abstract

A major issue in oncology is the high failure rate of translating preclinical results in successful clinical trials. Using a virtual clinical trial simulations approach, we present a mathematical framework to estimate the added value of combinatorial treatments in ovarian cancer. This approach was applied to identify effective targeted therapies that can be combined with the platinum-taxane regimen and overcome platinum resistance in high-grade serous ovarian cancer. We modeled and evaluated the effectiveness of three drugs that target the main platinum resistance mechanisms, which have shown promising efficacy *in vitro*, *in vivo*, and early clinical trials. Our results show that drugs resensitizing chemoresistant cells are superior to those aimed at triggering apoptosis or increasing the bioavailability of platinum. Our results further show that the benefit of using biomarker stratification in clinical trials is dependent on the efficacy of the drug and tumor composition. The mathematical framework presented herein is suitable for systematically testing various drug combinations and clinical trial designs in solid cancers.

## Introduction

The development of effective treatments for advanced solid cancers has turned out to be more difficult than expected, and the current success rate of translating encouraging preclinical results into successful clinical trials is low^1,2^. To increase the success rate, we and others have suggested using a virtual clinical trial (VCT) approach to systematically test the effectiveness of new treatment regimens^3–5^. The VCTs allow for the creation of a large *in silico* cohort of patients, whose collective response to a drug or drug combinations corresponds to real-life cohorts. A typical VCT has the advantage of taking longitudinal clinical, radiological, or preclinical data to rapidly estimate the effectiveness of treatment modalities.

High-grade serous ovarian cancer (HGSOC) is the most abundant and lethal epithelial ovarian cancer subtype, accounting for approximately 80% of the cases with a five-year survival rate of 43%^6^. The standard-of-care (SOC) therapy for metastasized HGSOC consists of debulking surgery and platinum-taxane chemotherapy. Even though most of the advanced HGSOC patients respond well to SOC, nearly all patients relapse and develop resistance to platinum-based chemotherapy. Thus, identifying effective therapies that can prevent the emergence or overcome platinum resistance would potentially improve the outcomes of patients with HGSOC.

Platinum resistance is a complex and multifactorial process^7,8^. While several mechanisms lead to platinum resistance, three main categories are^9,10^ (1) reduced intake or increased efflux of platinum, leading to reduced platinum bioavailability in a cell (pre-target resistance); (2) enhanced DNA repair mechanisms that overcome platinum-induced DNA adducts (on-target resistance); and (3) dysfunctional apoptosis machinery (post-target resistance). Targeting these major resistance mechanisms is an area of intensive research. For instance, the copper influx transporter (CTR1) is a primary platinum uptake transporter, whose high expression correlates with longer disease-free intervals and overall survival^11^. Preclinical data demonstrate that trientine, a copper chelating agent, increases platinum uptake through CTR1^12,13^, and early-phase clinical data show evidence that trientine may reverse platinum resistance^14^. Cell cycle regulation is a crucial step in efficient DNA repair^15^, and Wee1 inhibitors are emerging cell cycle inhibitors that have been successfully combined with platinum. Indeed, preclinical and early-phase clinical studies indicate that Wee1 inhibitors are able to resensitize platinum-resistant cells^15–17^. In addition, compounds targeting the apoptosis machinery have been suggested to overcome platinum resistance. Downregulation or pharmacological inhibition of the inhibitor of apoptosis protein (IAP) family with birinapant resulted in enhanced apoptosis *in vitro* and prolonged survival time in mice^18^. Furthermore, an early clinical trial suggested that a combination of platinum and birinapant is well-tolerated^19^.

Previously, we have shown that an HGSOC patient, on average, has five active platinum resistance mechanisms present already at diagnosis^5^. However, combination treatment where standard-of-care chemotherapy is supplemented with targeted drugs for the three most dominant resistant mechanisms would significantly prolong patient survival. Accordingly, we hypothesize here that an effective strategy to overcome platinum resistance is to combine platinum-based chemotherapy with drugs targeting the three major classes of platinum resistance mechanisms; pre-target, on-target, and post-target.

We have developed a mathematical framework to conduct virtual clinical trials (VCTs) during first-line treatment of HGSOC. We modeled novel combinations targeting three major (pre-target, on-target, and post-target) classes of platinum resistance, and show that a drug sensitizing resistant cells to platinum is superior to agents directly killing the resistant cells. Using VCT approach, we identify the most efficient combinations, and demonstrate that patient stratification with molecular biomarkers further improves patient outcomes.

## Results

### Mathematical model describes innate platinum resistance as a multifactorial process

**Figure 1:**
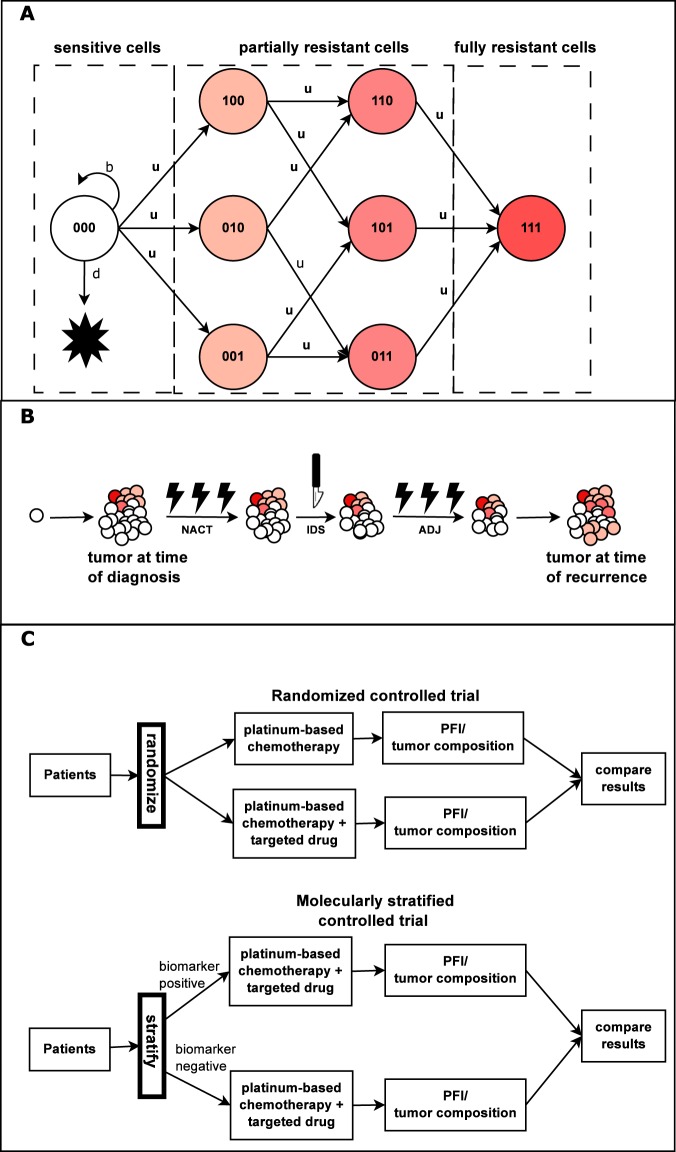
Schematics of the mathematical model and virtual clinical trial simulations framework. Panel (A) We developed a mathematical model that consists of three main platinum resistance mechanisms, pre-target, on-target, and post-target resistance. Accordingly, this gives rise to eight types of cancer cells that are referred to as sensitive (no resistance mechanisms active), partially resistant (one or two resistance mechanisms active), and fully resistant (all three resistance mechanisms active). All cancer cells proliferate at rate *b* and die at rate *d* (only shown for sensitive cells). Panel (B) The model simulation starts from a single sensitive cancer and the tumor progresses until diagnosis (pre-treatment phase). We modeled HGSOC patients undergoing neoadjuvant chemotherapy (NACT) standard-of-care (SOC) treatment for HGSOC, which consists of NACT followed by interval debulking surgery (IDS) and adjuvant chemotherapy (ADJ). After the treatment phase, the model is simulated until the first recurrence (post-treatment phase. Panel (C). The simulations contain randomized controlled trial simulations (RCTSs) and molecularly stratified controlled trial simulations (MSCTSs). An RCTS estimates the added value of a drug in a general HGSOC patient cohort and corresponds to traditional clinical trials where patients are not stratified based on a molecular biomarker. An MSCTS estimates the added value of using a molecular biomarker that stratifies patients according to their dominant drug resistance mechanism.

We constructed a multitype branching process mathematical model that describes the dynamics of platinum sensitive, partially sensitive, and fully resistant cells in advanced HGSOC. The model schematic is presented in Fig. 1A. Briefly, we included in the model three main platinum resistance mechanisms: reduced influx/increased efflux of platinum (pre-target), enhanced DNA damage response pathway (on-target), and damaged apoptosis machinery (post-target), which accumulate during cell division because of (epi)genetic aberrations. Detailed model description and all model assumptions are presented in the Materials and Methods section and Supplementary Text 1. All parameter values are listed in Table 1. The model simulation of each virtual HGSOC patient was performed in accordance with neoadjuvant chemotherapy (NACT) SOC in HGSOC meaning that two types of interventions are included in the simulations: interval debulking surgery and platinum-based chemotherapy. The model simulation is composed of three phases: pre-diagnosis, treatment, and post-treatment as shown in Fig. 1B.

**Table 1:** Standard parameters and their values for the platinum resistance mathematical model. “—” represents dimensionless unit.

Symbol	Value	Unit	Description	Reference
*b*	0.667	$\frac{1}{{\rm{day}}}$	Division rate	Taken from^27^
*d*	0.661	$\frac{1}{{\rm{day}}}$	Death rate	Taken from^29^
*u*	10^−5^	$\frac{1}{{\rm{cell}}\,{\rm{division}}}$	Transition rate	Taken from^5^
*M*	3.959·10^11^	# of cells	Average tumor burden at diagnosis	Taken from^5^
*M*_*relapse*_	10^9^	# of cells	Tumor burden at recurrence	Taken from^30^
*d*_*chemotherapy*_	0.856	—	Average chemotherapy effect on sensitive cells	Estimated from a calibration cohort from^5^
α_1_	0.02	—	Weight of chemotherapy effects on cells with one resistance mechanism	Estimated from a calibration cohort from^5^
α_2_	0.01	—	Weight of chemotherapy effects on cells with two resistance mechanisms	Estimated from a calibration cohort from^5^
β	2	cell-log kill	Fraction of cells removed by surgery	Taken from^5^

**Figure 2:**
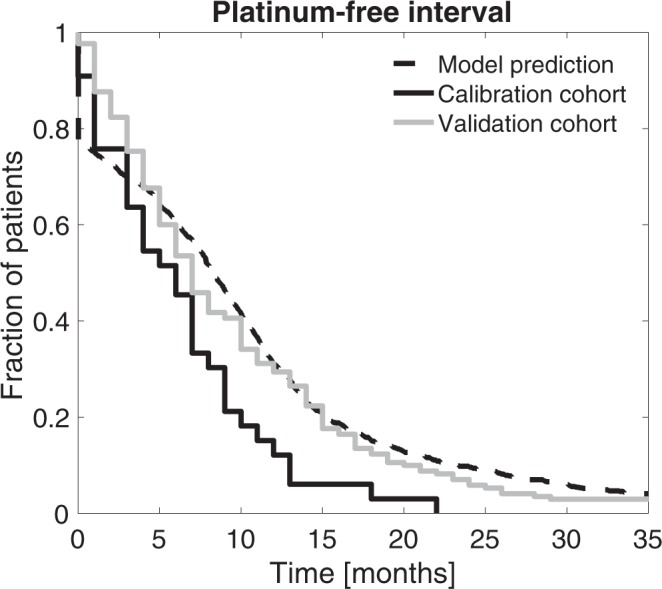
Model calibration. Estimated survival values for the calibration, validation, and simulated cohorts. Kaplan-Maier analysis was performed for the calibration and validation cohorts based on platinum-free interval (PFI) values. The mathematical model is capable of creating HGSOC cohorts that closely resemble real-life cohorts. Calibration of the targeted treatment is presented in Supplementary Fig. 2.

Response to platinum in HGSOC patients is clinically measured using platinum-free interval (PFI) defined as the time from last platinum therapy to tumor relapse^20^. Clinically, the first PFI predicts outcomes and dictates the second-line therapeutic approaches for the patients^21^. Next, we evaluated whether our model of innate platinum resistance was able to predict patient outcomes. As shown in Fig. 2, our model accurately estimated PFIs. The median PFI of patients in the calibration and validation cohorts equaled six and seven months, respectively, and the median PFI for the virtual cohort of 1,000 patients was eight months.

### Virtual clinical trial analysis estimates the added value of targeted therapy

We used our mathematical model and a VCT approach to evaluate the added value of combining drugs that target the three main resistance mechanisms to platinum. We created a virtual cohort of advanced HGSOC patients treated with platinum-based chemotherapy alone or a combination of platinum-based chemotherapy together with a drug targeting one of three platinum resistance mechanisms. Details of the targeted therapies included in the model are explained in Supplementary Text 1, and the VCT approach is schematically depicted in Fig. 1C. Model calibration is described in Supplementary Text 4. Model prediction with clinical data from the calibration and validation cohorts are shown in Fig. 2.

**Figure 3:**
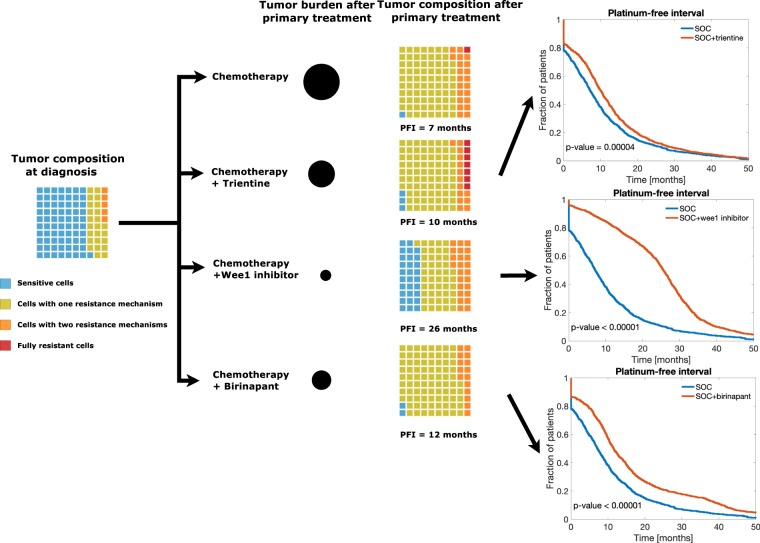
The added value of adding one platinum resistance targeting drug to platinum-taxane treatment. The waffle plots illustrate the fraction of sensitive, partially, and fully resistant cells in tumors. Black circles represent tumor burden after primary treatment relative to the tumor burden after platinum-based chemotherapy alone. The number below the waffle plots is the median PFI. Kaplan-Meier plots of PFI for each combination therapy (red line) compared to chemotherapy alone (blue line). The graphs were compared using log-rank test. Adding Wee1 inhibitor to platinum-based chemotherapy results in a PFI of 26 months, which is 19 months more than with the SOC. Furthermore, after adding Wee1 inhibitor, the residual tumor contains a sizable fraction of platinum-sensitive cells, which increases the probability of a successful platinum rechallenge in the subsequent treatment cycles. All two- and three-drug combination results are shown in Supplementary Fig. 3.

Our results show that at diagnosis, the tumor is mostly composed of sensitive cells and a small fraction of resistant cells (Fig. 3), which explains the observed good initial response in HGSOC. However, our results further indicate that after platinum-based chemotherapy, the tumor is mainly composed of cells with one active drug resistance mechanism, which undermines the effect of subsequent chemotherapy cycles. There results highlight the importance of tumor composition in chemotherapy effectiveness.

We then modeled the combination of platinum-based chemotherapy and trientine, Wee1 inhibitor, or birinapant as examples of drugs targeting the different resistance mechanisms and estimated their effects on PFI and tumor composition (Fig. 3). Combining platinum and trientine led to a 99% decrease in tumor burden and a PFI of three months longer in comparison to platinum-based chemotherapy alone (p = 0.00004, log-rank test). However, trientine caused an enrichment of fully resistant cells compared to the other drugs, which suggests that platinum rechallenge after trientine may not have a significant impact. The addition of birinapant decreased tumor burden by 99% and improved the PFI by five months (p < 0.00001, log-rank test). The biggest decrease in tumor burden (99.99%) and improvement in PFI (19 months, p < 0.00001, log-rank test) was obtained when Wee1 inhibitor was combined with platinum. Importantly, combining Wee1 inhibitor to chemotherapy led to a larger retainment of platinum-sensitive cancer cells (29%) compared to the other combination therapies (3% and 2% for trientine and birinapant, respectively), consistent with the PFI benefit from this combination.

The results from combining simultaneously two and three drugs are presented in Supplementary Fig. 3. Briefly, the best combination of two targeted drugs with the platinum-based chemotherapy was Wee1 inhibitor and birinapant, which led to a median PFI of 56 months. Birinapant combined with trientine led to the shortest median PFI of 17 months. When combining all three drugs with platinum, the median PFI increased to 59 months, and approximately 85% of the remaining cancer cells were sensitive to platinum-based chemotherapy.

The effect of targeting the dominant platinum resistance mechanism depends on drug efficacy and tumor composition. The effect of a targeted drug is hypothesized to be improved when the choice of a drug is guided using a molecular biomarker. We tested this hypothesis using the MSCTS approach.

**Figure 4:**
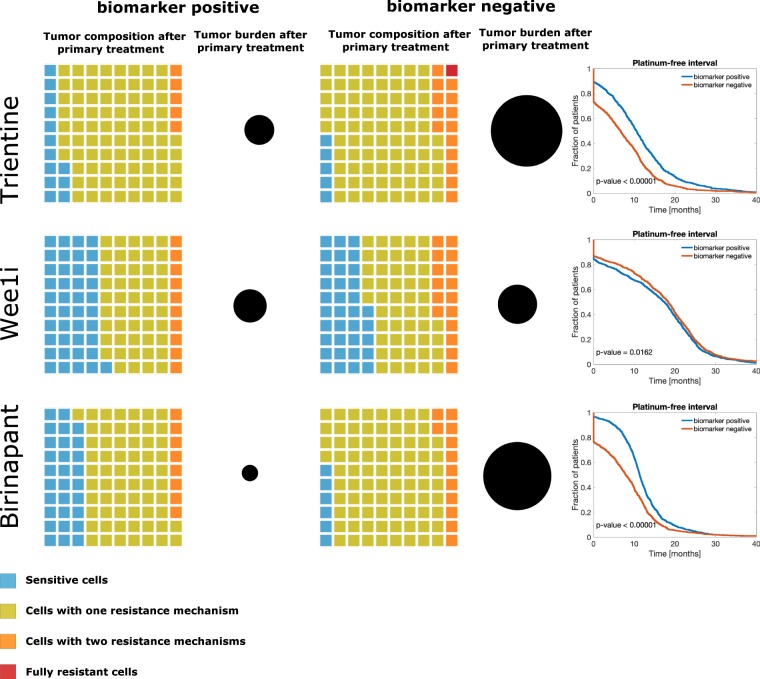
Molecularly stratified clinical trials simulations. The waffle plots illustrate tumor composition after primary treatment and the black circle the tumor burden. In the right column, Kaplan-Meier plots showing the comparison of the biomarker-positive and biomarker-negative VCT arms.

We tested the benefit of choosing one targeted drug based on a biomarker for the dominant resistance mechanism and combining it with the platinum-based chemotherapy (Fig. 4 and Table 2). The benefit of choosing patients positive to trientine was four months in comparison to patients without the positive marker (p < 0.0001, log-rank test). For birinapant, the relative PFI benefit was three months. Thus, adding biomarker-based patient stratification was highly beneficial for trientine and birinapant (p < 0.00001, log-rank test). For Wee1 inhibitor, the relative PFI benefit was one month (18 months vs. 17 months, p = 0.02, log-rank test). However, combining Wee1 to chemotherapy in a biomarker-unselected population was still superior to the biomarker-selected treatments combining trientine (p < 0.0001) or birinapant (p < 0.0001) with chemotherapy.

**Table 2:** Molecularly stratified clinical trial simulations.

Drug	Biomarker negative	Biomarker positive	Relative benefit
Trientine	7	11	4
Wee1 inhibitor	17	18	1
Birinapant	8	11	3

Median PFI values for two treatment arms in MSCTS and relative benefit of patient stratification. The values are presented in months.

## Discussion

A major reason for the low success rate in translating preclinical results into successful clinical trials is that *in vitro* and *in vivo* models rarely capture the variations in treatment responses seen in real-life patient cohorts. Here, we have developed a mathematical model to create virtual patient cohorts that enabled us to test the effectiveness of targeted therapy to overcome platinum resistance. Our framework consists of (1) a stochastic model of innate platinum resistance, (2) a method for the estimation of relative drug efficacy based on three levels of evidence, (3) a model of targeted therapy, and (4) a simulator of virtual clinical trials.

In our approach, we did not explicitly consider toxicity when combining the drugs, which will need additional clinical consideration. Thus, in our case study we chose targeted drugs that have been well tolerated in combination with platinum in clinical trials. Furthermore, we focused on modeling cancer cell evolution and response to chemotherapy, and interaction with the tumor microenvironment, which plays an important but poorly understood role in chemoresistance, will be conducted in future work. The weighting of evidence from *in vitro* experiments, *in vivo* experiments and clinical trial experiments for drug efficacy is based on the fact that only a small fraction of drugs that successfully eradicate tumor cells *in vitro* make it to the clinical trials and only 1 out of 50 drugs entering clinical trials are successful, which indicates that the data arising from preclinical models have a lower likelihood of success in clinical translation. The evidence weights can be modified whenever new preclinical or clinical data arise on single-agent therapies.

We tested our framework by estimating the added value of combining platinum with trientine, Wee1 inhibitor, and birinapant. Our results indicate that targeting one resistance mechanism with platinum-based chemotherapy increases PFI by several months. The most effective drug with platinum-taxane was Wee1 inhibitor, which tripled the PFI as compared to SOC. Moreover, the residual tumor after Wee1 inhibitor consisted of a large fraction of platinum-sensitive cancer cells, which predicts that the subsequent platinum-based chemotherapy cycles will be effective. Our results suggest that the class of targeted drugs that resensitize chemoresistant cells is more effective than drugs aimed at triggering apoptosis or increasing the bioavailability of platinum. This is in line with promising results from the use of Ataxia telangiectasia and Rad3-related (ATR) inhibitors in resensitizing platinum-resistant cancers^22^.

Molecular biomarkers are increasingly used to stratify patients in clinical trials, such as in SHIVA and NCI-MATCH^23,24^. We used the modeling framework to estimate the benefit of using molecular markers in stratifying patients. Our results suggest that the two key factors affecting the survival benefit of a stratified trial are the efficacy of the drug and tumor composition. The most potent drug in our simulation, Wee1 inhibitor, had virtually identical effects in stratified and non-stratified patient cohorts, whereas trientine and birinapant benefited from stratification. The reason for this is that the total relative efficiency of the Wee1 inhibitor is estimated to be more than two times greater than that of trientine and birinapant. Thus, the Wee1 inhibitor very effectively kills cancer cells with an active on-target resistance mechanism. Although an on-target resistance mechanism may not be dominant in many cases, its fraction is still so large that the Wee1 inhibitor remains a very potent option even in a biomarker-unselected patient population. In contrast, in the less potent trientine and birinapant combinations, tumor-free time was clearly improved with patient stratification. This indicates that in the biomarker stratified trials, the estimated effect of the tested drug on the tumor composition should be, if possible, taken into account in the trial planning phase.

The herein presented mathematical framework is suitable for systematically testing various drug combinations and clinical trial designs in solid cancers. The major advantage of the framework is that it enables estimating the efficacy of a drug combination in an inexpensive and rapid manner, and thus facilitates the design of combination-therapy clinical trials.

## Methods

### Description of calibration and validation cohort

Clinical data for HGSOC patients were collected as explained in^5^. Briefly, the first cohort included 62 advanced stage III–IV HGSOC patients treated at Turku University Central Hospital from 2009 to 2016 and was used as a calibration cohort. The patients participating in the study gave their informed consent, and the study and the use of all clinical material have been approved by (i) The Ethics Committee of the Hospital District of Southwest Finland (ETMK): ETMK 53/180/2009 × 238 and (ii) National Supervisory Authority for Welfare and Health (Valvira): DNRO 6550/05.01.00.06/2010 and STH507A, and all experiments were performed in accordance with the guidelines and regulations. All patients in this cohort were evaluated as inoperable at the time of diagnosis and were referred to neoadjuvant chemotherapy (NACT) before interval debulking surgery (IDS).

To reliably estimate the effects of chemotherapy in HGSOC patients, we calculated total metabolic tumor volume (MTV) as the sum volume of all the tumor lesions from ^18^F-FDG-PET/CT images before and after NACT^5^. This change in tumor burden was used for the model construction and calibration. The second cohort was used as an independent validation cohort, and it included 170 patients from The Cancer Genome Atlas (TCGA). The validation cohort was filtered from a cohort of 489 HGSOC patients available in TCGA, using similar criteria for patient characteristics as in the calibration cohort, including patients with (i) advanced HGSOC (grade >1, stage: IIIb–IV), (ii) an indication of attempted surgical debulking, that is, information available on the residual disease, and (iii) treatment by platinum-based chemotherapy alone or in combination in the first-line setting (for more detailed explanation see^5^). We used platinum-free interval (PFI), defined as the time from last platinum-based chemotherapy to tumor relapse as the primary outcome measure, and secondary outcome measures were tumor burden and its composition.

### Mathematical model of innate resistance to platinum and targeted therapy

Our mathematical model describes tumor growth and the evolution of platinum resistance as a multitype branching process^25,26^. Innate platinum resistance is a multifactorial process. According to the mechanisms of action, it can be broadly divided into three main classes: pre-target, on-target, and post-target resistance. The model includes these three main classes of drug resistance mechanisms. The schematic of the model is shown in Fig. 1A, where each subclone is defined with three digits: 0 (1) mean absence (presence) of given platinum resistance mechanism.

Most of the parameter values are taken from previous studies^5,27^, whereas the parameters *u*, *α*_1_, and *α*_2_ were fitted to the clinical data from the calibration cohort as described in the Supplementary Text 4. In short, we took 100 values for *u* between 10^−4^ to 10^−8^ and 101 values for *α*_1_ and *α*_2_ between 0 and 1, leading to 1,020,100 parameter combinations. Next, we performed a grid search by performing a PFI analysis for each parameter combination, and we checked the agreement of the model predictions with the clinical data from the calibration cohort by calculating the root-mean-square error (RMSE). Finally, we chose the combination of *u*, *α*_1_, and *α*_2_ that had the smallest RMSE.

In addition to modeling innate platinum resistance, we also modeled targeted therapy. That is, we included three drugs: trientine, Wee1 inhibitor, and birinapant which target pre-, on- and post-target platinum resistance respectively. Details of our mathematical model and inclusion of targeted therapy is described in Supplementary Text 1 and presented schematically in Supplementary Fig. 1. The model was implemented and simulated using MATLAB environment. The code of the model simulator is freely available (https://github.com/EmiliaKo/vctsInOVCA).

### Drug efficacy estimation for combinatorial treatment analysis

To estimate the relative efficacy (*E*) of targeted therapy, we searched and evaluated the relative efficacies in comparison to platinum alone reported in the literature at three levels of evidence: *in vitro* (*I*), *in vivo* (*V*), and clinical trial (*C*). We calculated the efficacy of the targeted therapies in relation to platinum. Thus, a drug with 100% relative efficacy has an identical effect as platinum.

The relative efficacy of a drug is extracted from the literature as a reported reduction of tumor burden in comparison to platinum-based chemotherapy. As *in vitro* drug efficacy is often higher than observed *in vivo* or clinical trials, we estimated a drug’s relative efficacy as follows. The relative efficacy of a drug in a clinical trial is considered 2.5 times more important than *in vitro*, and *in vivo* relative efficacy is weighted as 60% of the evidence from clinical trials. Thus, total relative efficacy (*E*) is calculated as:

$${E}=0.2\cdot {I}+0.3\cdot {V}+0.5\,{C}$$

The values for *E*, *I*, *V*, and *C* are listed in Supplementary Table 1. The relative efficacies are used in the model as a relative reduction in tumor burden after primary treatment in comparison to platinum-based chemotherapy alone. The estimated total relative efficacy for trientine was 41%, for Wee1 inhibitor 85%, and for birinapant 35%.

### Randomized and molecularly stratified virtual clinical trial simulations

We designed two types of VCTs: a randomized controlled trial simulation (RCTS) without molecular marker-guided patient selection and a molecularly stratified controlled trial simulation (MSCTS). We first performed a RCTS to estimate the effectiveness of the targeted therapies in non-selected HGSOC patients. Next, we performed a MSCTS to assess the putative improvement of PFI when a biomarker for the dominant resistance mechanism was used to select patients.

Both types of clinical trial simulations were performed by simulating the course of tumor progression (Fig. 1B). In addition, primary treatment was simulated according to the SOC in HGSOC. That is, simulation of the treatment phase was performed according to National Comprehensive Cancer Network (NCCN) guidelines, which suggest platinum-taxane chemotherapy and debulking surgery as the first-line treatment^28^. Before starting the simulations, a virtual patient was defined with two model parameters (*M*_*diagnosis*_ and *d*_*chemotherapy*_) and sampling them from the log-normal probability distribution. Next, the model simulation started with a single sensitive cell until the time of diagnosis, which is defined as the time when tumor burden reaches *M*_*diagnosis*_ cells (pre-treatment phase). After diagnosis, primary treatment composed of three cycles of NACT, IDS, and three cycles of adjuvant chemotherapy (ADJ) was administered (treatment phase). The model was simulated until the first relapse, and PFI was used as the outcome (post-treatment phase).

The RCTS was performed by assigning virtual randomly selected HGSOC patients to one of two groups: platinum-based chemotherapy alone or platinum-based chemotherapy with a drug targeting a platinum resistance mechanism. Next, each virtual HGSOC patient in the cohort was simulated. To each of the groups in the RCTS, we assigned 1,000 virtual patients, leading to a total cohort of 2,000 virtual patients. Finally, patient’s outcome was measured using PFI. In addition, the tumor composition after primary treatment was measured and compared.

In the MSCTS, virtual patients were stratified using molecular biomarkers. Here, we assumed that a reliable biomarker existed for each of the platinum resistance mechanisms included in the model. In the simulations, we assumed that the biomarkers were ideal (i.e., their sensitivities and specificities were 100%). We used the biomarkers as follows. First, 1,000 virtual patients were assigned to the biomarker positive and the biomarker negative groups, according to the dominant mechanisms. For instance, for Wee1 inhibitor, the biomarker positive group consisted of patients with on-target platinum resistance as the dominant resistance mechanism, while the biomarker negative group contained patients with pre-target or post-target resistance as their dominant mechanisms. The patients in both groups were treated with platinum-based chemotherapy and Wee1 inhibitor, followed by Kaplan-Meier analysis and average tumor composition.

## Supplementary information

Supplementary materials

## Data Availability

All data generated or analyzed during this study are included in this published article (and its Supplementary Information files). The model simulator is available in GIThub repository (https://github.com/EmiliaKo/vctsInOVCA).

## References

[1] Lieu CH, Tan A-C, Leong S, Diamond JR, Eckhardt SG (2013). From bench to bedside: lessons learned in translating preclinical studies in cancer drug development. JNCI J. Natl. Cancer Inst..

[2] Kim E, Rebecca VW, Smalley KSM, Anderson ARA (2016). Phase i trials in melanoma: A framework to translate preclinical findings to the clinic. Eur. J. Cancer.

[3] Harrison RL (2014). A virtual clinical trial of FDG-PET imaging of breast Cancer: effect of variability on response assessment. Transl. Oncol..

[4] Han K (2016). Simulations to predict clinical trial outcome of Bevacizumab Plus chemotherapy vs. chemotherapy alone in patients with first-line gastric cancer and elevated plasma VEGF-A. CPT Pharmacometrics Syst. Pharmacol..

[5] Kozłowska E (2018). Mathematical Modeling Predicts Response to Chemotherapy and Drug Combinations in Ovarian Cancer. Cancer Res..

[6] Torre LA (2018). Ovarian cancer statistics, 2018. CA. Cancer J. Clin..

[7] Colombo P-E (2014). Sensitivity and resistance to treatment in the primary management of epithelial ovarian cancer. Crit. Rev. Oncol. Hematol..

[8] Tapia Gonzalo, Diaz-Padill Ivan (2013). Molecular Mechanisms of Platinum Resistance in Ovarian Cancer. Ovarian Cancer - A Clinical and Translational Update.

[9] Galluzzi L (2014). Systems biology of cisplatin resistance: past, present and future. Cell Death Dis..

[10] Galluzzi L (2012). Molecular mechanisms of cisplatin resistance. Oncogene.

[11] Kilari D, Guancial E, Kim ES (2016). Role of copper transporters in platinum resistance. World J. Clin. Oncol..

[12] Liang ZD (2012). Mechanistic basis for overcoming platinum resistance using copper chelating agents. Mol. Cancer Ther..

[13] Brown DPG, Chin-Sinex H, Nie B, Mendonca MS, Wang M (2009). Targeting superoxide dismutase 1 to overcome cisplatin resistance in human ovarian cancer. Cancer Chemother. Pharmacol..

[14] Fu S (2014). Exploratory study of carboplatin plus the copper-lowering agent trientine in patients with advanced malignancies. Invest. New Drugs.

[15] Hirai H (2009). Small-molecule inhibition of Wee1 kinase by MK-1775 selectively sensitizes p53-deficient tumor cells to DNA-damaging agents. Mol. Cancer Ther..

[16] Osman AA (2015). Wee-1 Kinase Inhibition Overcomes Cisplatin Resistance Associated with High-Risk TP53 Mutations in Head and Neck Cancer through Mitotic Arrest Followed by Senescence. Mol. Cancer Ther..

[17] Leijen S (2016). Phase II study of Wee1 inhibitor AZD1775 plus carboplatin in patients with tp53-mutated ovarian cancer refractory or resistant to first-line therapy within 3 months. J. Clin. Oncol..

[18] Janzen DM (2015). An apoptosis-enhancing drug overcomes platinum resistance in a tumour-initiating subpopulation of ovarian cancer. Nat. Commun..

[19] Noonan AM (2016). Pharmacodynamic markers and clinical results from the phase 2 study of the SMAC mimetic birinapant in women with relapsed platinum-resistant or -refractory epithelial ovarian cancer. Cancer.

[20] Colombo N (2014). Optimising the treatment of the partially platinum-sensitive relapsed ovarian cancer patient. Eur. J. Cancer, Suppl..

[21] Plaxe, S. C. *et al*. *NCCN Guidelines Ovarian Cancer Including Fallopian Tube Cancer and Primary Peritoneal Cancer*. (2017).

[22] Mei L, Zhang J, He K, Zhang J (2019). Ataxia telangiectasia and Rad3-related inhibitors and cancer therapy: where we stand. J. Hematol. Oncol..

[23] Tourneau, C. L. *et al*. Molecularly targeted therapy based on tumour molecular profi ling versus conventional therapy for advanced cancer (SHIVA): a multicentre, open-label, proof-of-concept, randomised, controlled phase 2 trial. **16** (2015). 10.1016/S1470-2045(15)00188-6 26342236

[24] Barroilhet L, Matulonis U (2018). The NCI-MATCH trial and precision medicine in gynecologic cancers. Gynecol. Oncol..

[25] Durrett Richard (2015). Branching Process Models of Cancer. Branching Process Models of Cancer.

[26] Kimmel, M. & Axelrod, D. E. *Branching Processes in Biology*. **19**, (Springer-verlag New York, 2002).

[27] Panetta JC (1997). A mathematical model of breast and ovarian cancer treated with paclitaxel. Math. Biosci..

[28] Armstrong DK (2018). New Therapies for Ovarian Cancer. J. Natl. Compr. Cancer Netw..

[29] Brown PO, Palmer C (2009). The preclinical natural history of serous ovarian cancer: Defining the target for early detection. PLoS Med..

[30] Del Monte U (2009). Does the cell number 109 still really fit one gram of tumor tissue?. Cell Cycle.

