# Author Correction: Virtual clinical trials identify effective combination therapies in ovarian cancer

**DOI:** 10.1038/s41598-020-69092-x

**Published:** 2020-07-22

**Authors:** Emilia Kozłowska, Tuulia Vallius, Johanna Hynninen, Sakari Hietanen, Anniina Färkkilä, Sampsa Hautaniemi

**Affiliations:** 1 Research Program in Systems Oncology, Research Programs Unit, Faculty of Medicine, University of Helsinki, Helsinki, Finland; 2 Institute of Automatic Control, Silesian University of Technology, Akademicka 16, 44-100 Gliwice, Poland; 3 Department of Oncology and Radiotherapy, Turku University Hospital, University of Turku, Turku, Finland; 4 Department of Obstetrics and Gynecology, Turku University Hospital, University of Turku, Turku, Finland; 5 Department of Radiation Oncology, Dana-Farber Cancer Institute, Harvard Medical School, Boston, USA; 6 Department of Obstetrics and Gynecology, Helsinki University Hospital, Helsinki, Finland

Correction to: *Scientific Reports* 10.1038/s41598-019-55068-z, published online 10 December 2019

This Article contains a typographical error in the Acknowledgements section.

“This project has received funding from the European Union’s Horizon 2020 Research and Innovation Programme under grant agreement No. 66740, the Academy of Finland, the Sigrid Jusélius Foundation, Finnish Cancer Associations and by National Science Centre, Poland (http://ncn.gov.pl), grant DEC 2016/21/B/ST7/02241. The results published here are in part based upon data generated by TCGA managed by the NCI and NHGRI. Information about TCGA can be found at http://cancergenome.nih.gov.”

should read:

“This project has received funding from the European Union’s Horizon 2020 Research and Innovation Programme under grant agreement No. 667403 for HERCULES, the Academy of Finland, the Sigrid Jusélius Foundation, Finnish Cancer Associations and by National Science Centre, Poland (https://ncn.gov.pl), grant DEC 2016/21/B/ST7/02241. The results published here are in part based upon data generated by TCGA managed by the NCI and NHGRI. Information about TCGA can be found at https://cancergenome.nih.gov.”

