# Supplementary material for: Virtual clinical trials identify effective combination therapies in ovarian cancer
Source: Sci Rep. 2019 Dec 10;9:18678. doi: 10.1038/s41598-019-55068-z (PMC6904444; doi:10.1038/s41598-019-55068-z)
Supplement: Supplementary file 1 — Supplementary materials [file 41598_2019_55068_MOESM1_ESM.pdf]

Supplementary material: Virtual clinical trials identify effective combination therapies in ovarian cancer

Emilia Kozłowska, Tuulia Vallius, Johanna Hynninen, Sakari Hietanen, Anniina Färkkilä, Sampsa Hautaniemi

Text S1 Mathematical model

Model structure

In this section, details of the mathematical model are presented. The model describes tumor growth and the evolution of platinum resistance as the multitype–type discrete–state continuous–time branching process model. The model structure is illustrated in Figure 1.

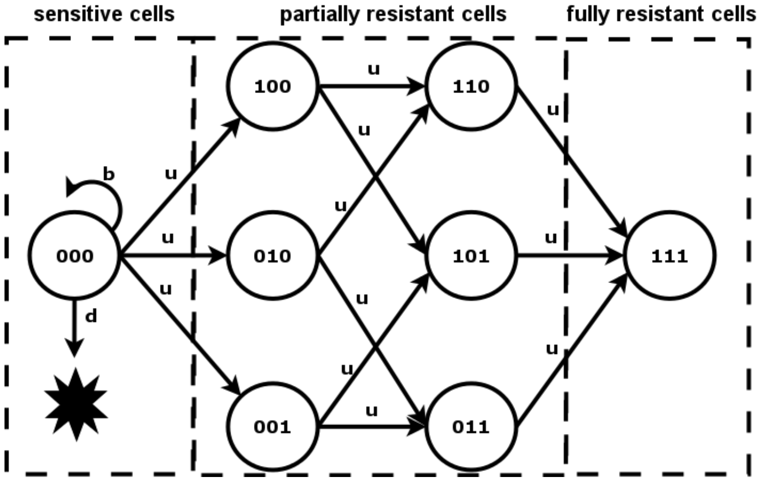

Figure 1: Illustration of the the mathematical model structure. The model includes eight type of cells, which are defined with a binary string of size three as the model consists of three platinum resistance mechanisms. The numbers 0(1) mean absence (presence) of a given platinum resistance mechanism in the cell.

In the model, every cell can undergo one of three processes: i) faithful division where new cell with

the same phenotype emerges, ii) unfaithful division where during division new cell type appear with rate u per cell division, and iii) death where cell dies and is removed from the system.

We have included three main platinum resistance mechanisms (pre–target, on–target and post–target). As we consider all possible cell types, the model with three platinum resistance mechanisms contains eight subclones: 000, 100, 010, 001, 101, 110, 011, 111, where 0(1) indicates the presence (absence) of a platinum resistance mechanism. The first, the second and the third digit indicates the presence (absence) of pre-target, on-target, and post-target platinum resistance, respectively. In the absence of treatment intervention, all cells grow exponentially with the same birth rate b. It means that we consider the evolution of platinum resistance as neutral. In addition, all cells die with death rate d leading to net growth rate $\lambda = b - d$. In the presence of chemotherapy treatment, however, sensitive cells (000) die with additional rate $d_{chemotherapy}$ proportionally to birth rate leading to new death rate $d' = d + b \cdot d_{chemotherapy}$. Fully resistant cells are not affected by chemotherapy, *i.e.*, $d_{chemotherapy} = 0$. Cells with one and two platinum resistance mechanisms have $d_{chemotherapy}$ decreased by α_1 and α_2, respectively. Thus, in the presence of treatment intervention, partially and fully resistant cells have selection advantage over sensitive ones.

In addition to the faithful division, where division leads to the occurrence of the second cell with the same phenotype, a cell can divide where new subclone with an additional resistance mechanism appears. In the model, we do not take into account cross–resistance, and as a result, during one division, only one additional platinum resistance mechanism can be acquired. In addition, we also assume that the acquisition of platinum resistance mechanism is irreversible, leading to transitions between all eight cell types, as shown in Figure 1. As innate resistance is known as the dominant one, and acquired resistance has a minor effect, transition rate u is the same with or without chemotherapy and targeted therapy treatment intervention.

In the model, we also included three types of treatment interventions: debulking surgery, platinum-based chemotherapy, and targeted treatment. Surgery is modeled as the removal of a fraction of β cancer cells, and all cell types can be eliminated by the surgery with an equal probability. Chemotherapy is modeled by increasing the death rate of sensitive and partially resistant cells proportionally to the division rate. In the model, chemotherapy is administered as a finite time-continuous treatment with maximum tolerated dose (MTD), which is the current standard-of-care in high-grade serous ovarian cancer (HGSOC). Targeted treatment is modeled in combination with platinum-based chemotherapy. The details of inclusion of targeted treatment in the model are presented in the next section.

Modeling targeted treatment

Here, we focus on modulators of platinum resistance, which sensitize platinum-resistant cells to platinum-based chemotherapy. As in the model we consider three platinum resistance mechanisms; we included three classes of modulators where each one target different platinum resistance mechanism. The first class modulates platinum transport by increasing platinum concentration in the cell and thus targets pre–target platinum resistance. The second class regulates DNA damage response (DDR) by inhibition of the efficient DNA repair machinery in platinum-resistant cells. Thus, this class of modulators targets on–target platinum resistance. The last class of platinum sensitivity modulators targets post–target platinum resistance which is responsible for enhancing apoptosis rate.

One the most promising method to overcome platinum resistance is a combination of platinum-based chemotherapy with drugs targeting given platinum resistance mechanism [6]. In addition, the majority of the HGSOC patients have subclones that confer resistance to platinum at the time of diagnosis. These lead to the hypothesis that the best strategy to overcome platinum resistance would be a combination of platinum-based chemotherapy with a targeted therapy that hits the dominant resistance mechanisms as the first-line treatment.

Trientine

Cooper transporter receptor 1 (CTR1) is a major copper influx transporter that mediates transport of platinum to the cells. Clinical studies demonstrated that expression of CTR1 correlates with intratumor platinum concentration leading to better clinical outcome [10]. Copper-lowering agents, such as trientine, increase the expression of human CTR1 (hCTR1), which leads to resensitization of tumor cells to platinum [5].

In the model, trientine is included by increasing simultaneously two parameters α_1 and α_2 in partially resistant cells which are also pre-target resistant to platinum, *i.e.*, 100, 110, and 101. The parameter α_1 and α_2 defines how much chemotherapy killing effect is reduced in partially resistant cells in comparison to sensitive cells. As trientine increases platinum accumulation resulting in enhanced killing effect of platinum-based chemotherapy in pre–target resistant cells.

Wee1 inhibitor

An essential mechanism of action of platinum is introducing interstrand crosslinks to DNA, which need to be recognized and repaired by the DNA repair machinery. One important platinum resistance mechanism is the upregulation of the DNA damage response to cope with the increased replication stress induced by platinum. Preclinical and early clinical studies indicate that cell cycle checkpoint inhibitors, such as Wee1 inhibitors, sensitize platinum-resistant cells to platinum-based chemotherapy. The predominant mechanism-of-action of Wee1 inhibitors is to cause the failure of G2-M checkpoint due to inappropriate CDK1/cyclin B complex activation resulting in mitotic catastrophe.

In the model, we included Wee1 inhibitors by adding a reverse transition from fully-resistant to partially resistant cells and from partially resistant cells to the sensitive cell. There is a probability $u_{reverse}$ per cell division that the cell will reverse back from fully resistant cell to partially resistant cell and from partially resistant cell to sensitive cell (*e.g.*, from x011 to x010). Wee1 inhibitor has no single-agent activity in primary platinum-sensitive tumors [12]. Thus, we assume that Wee1 inhibitor does not kill platinum-resistant cells, but only sensitize cancer cells to platinum chemotherapy by reversion transition.

Birinapant

Cancer cells can resist programmed cell death, for example, by overexpression of proteins blocking pro-apoptotic pathways. Inhibition of apoptosis is a mechanism of platinum resistance, and thus activation of cancer cell death through apoptosis is an important process to resensitize cells to platinum-based chemotherapy. Upregulation of the gene inhibitor of apoptosis (IAP) is one mechanism leading ti apoptosis evasion. Thus, inhibition of IAP by an IAP inhibitor, such as birinapant, could sensitize cancer cells to platinum by activation of cancer cell death.

We included birinapant in the model by adding additional death with the rate d_a called apoptotic cell death. Thus, cells with active post–target platinum resistance have death rate equal to $d' = d + b \cdot d_{chemotherapy} + d_a$ during treatment phase. As a result, cancer cells with active post-target platinum resistance mechanism have a higher death rate than platinum-sensitive ones.

Key assumptions of the model

1. Only patients who are unresectable at the time of diagnosis are considered in the model simulations.
2. Cells are growing exponentially with net growth rate $\lambda = b - d$.

3. Platinum-sensitive cells can acquire one of three platinum resistance mechanism with equal probability of u per cell division.

4. During each cell division, sensitive or partially-resistant cells can acquire only one platinum resistance mechanism, leading to combinatorial network describing transitions between subclones as shown in Figure 1.

5. In the model, only innate platinum resistance is considered.

6. The platinum-resistant cells grow at the same rate as wild–type cells, *i.e.*, they are neutral in the absence of treatment.

7. Cancer is diagnosed when the colony reaches a certain size (M).

8. Rate of accumulation of platinum resistance in the absence of treatment is the same as during the treatment, *i.e.*, u is the same in all three phases of simulations.

9. Two types of treatment modalities (debulking surgery and platinum-based chemotherapy) are included in standard-of-care simulations.

10. The effect of chemotherapy is modeled by the drug–induced death rate, $d_{chemotherapy}$, which leads to negative net growth rate: $\lambda = b - d < 0$.

11. Chemotherapy toxicity is not considered in the current model implementation.

12. Chemotherapy administration is included in the model as a continuous-time maximum tolerated dose (MTD).

13. Surgery is modeled by the removal of a fraction of β cancer cells. All cell types can be removed with an equal probability.

14. Tumor recurrence is observed when the number of cancer cells reaches $M_{relapse}$ after end of primary treatment.

Computer simulation

We performed Monte Carlo computer simulation using an approximation of an exact stochastic simulation algorithm developed by Gillespie [7]. We used an approximation algorithm due to its computational efficiency. First, slow events (unfaithful division) are separated from fast events (faithful division, cell death). The propensities of slow reactions are: $a_i(x)$, for $i = 1, 2 \ldots n$, where n is the number of slow events. The propensities of fast reactions are: $b_i(x)$ for $j = 1, 2 \ldots m$, where m is the number of fast events. Next, time of the next slow reaction is calculated:

$$\tau = \frac{1}{\sum_{i=1}^{n} a_i(x)} \ln(\frac{1}{R_1}), \tag{S1}$$

where R_1 is a random number from a unit-interval uniform distribution. Next, the index of next slow event is given as the smallest k satisfying:

$$\sum_{k=1}^{n} a_k(x) > R_2 \cdot \sum_{i=1}^{n} a_i(x), \tag{S2}$$

where R_2 is a random number from a unit-interval uniform distribution. The system is then updated and fast events that occur until time of the first slow event τ. Fast events are updated using tau-leaping, *i.e.,* for every fast event the number of times each event occurs during the time interval $[t, t + \tau)$ is:

$$k_j = Poisson(a_j \cdot \tau) \quad , for\ j = 1, 2, \ldots, m \tag{S3}$$

and the system is updated:

$$x(t + \tau) = x(t) + \sum_j k_j \cdot v_j, \tag{S4}$$

where v_j is the state change vector.

Text S2 Standard-of-care simulations

To investigate resistance to platinum-based chemotherapy and suggest novel methods to resensitize HGSOC patients to standard-of-care treatment, we performed computer simulations of the model. The simulation consists of three phases: 1) pre–treatment phase in which the tumor grows until diagnosis, 2) treatment phase that includes platinum-based chemotherapy and debulking surgery, and 3) post–treatment phase where the tumor grows until the first relapse.

The first phase starts with a single platinum–sensitive cell and proceeds with exponential tumor growth in the absence of treatment interventions. In addition to the growth of the platinum–sensitive cells, accumulation of platinum resistance mechanisms is also simulated by the appearance and the growth of partially and fully resistant clones during unfaithful cell division. The phase ends when the total number of tumor cells reaches M cells. The tumor is then diagnosed, and the treatment phase starts.

Treatment phase starts after the patient is diagnosed with HGSOC, *i.e.*, we assume no delay in treatment initiation. Standard-of-care includes two types of interventions: platinum-based chemotherapy and debulking surgery. Chemotherapy reduces the net growth rate of sensitive and partially–resistant cells. As in the model, we do not consider pharmacokinetics and pharmacodynamics of platinum-based chemotherapy; we simulate platinum-based chemotherapy as a continuous injection. Debulking surgery is modeled by removing a fraction of tumor cells at a one-time point. Every cell can be removed during debulking surgery with the same probability. The treatment phase is simulated in a framework that follows the first-line standard-of-care therapy guidelines for HGSOC. Thus, in our simulations, the HGSOC patient is treated with three cycles of neoadjuvant chemotherapy (NACT). Next, a debulking surgery is performed, aiming for optimal cytoreduction, followed by another three cycles of adjuvant chemotherapy.

The last phase of simulation starts after the treatment phase and ends at the time of the first recurrence. In this phase of simulation, the tumor grows without treatment intervention until the total number of cells reaches $M_{relapse}$ cells.

Text S3 Simulation of virtual HGSOC patients cohort

We performed *in silico* treatment response analysis using the simulation approach described in Supplementary Text 2. Each virtual HGSOC patient is described with two parameters: tumor burden at diagnosis M and chemotherapy effect $d_{chemotherapy}$.

For each virtual HGSOC patient, we sampled tumor burden at diagnosis (M) and chemotherapy effect ($d_{chemotherapy}$) from a log–normal distribution. The log–normal distribution was selected because it describes the best distributions for M and $d_{chemotherapy}$ among six tested statistical distributions (details in Supplementary Text S4). The goal of sampling is to reproduce *in silico* real-life patient's heterogeneity of response to standard-of-care treatment.

Next, we simulated each virtual HGSOC starting from a single platinum-sensitive cell until the first relapse. The goal of the simulations was to compute the platinum-free interval from virtual HGSOC patients as well as the proportion of all cancer cells subclones at the time of diagnosis and after first-line treatment.

In our analysis, we perform simulation of 1,000 HGSOC patients to estimate response to standard-of-care treatment. The computed PFI estimates from virtual HGSOC patients were applied to perform Kaplan–Meier analysis. As a result, we created platinum–free interval survival plots. Kaplan–Meier analysis was carried out in MATLAB using ecdf function with no censoring as all virtual HGSOC patients relapse after first-line treatment.

Text S4 Parameter calibration

Tumor growth

The average cell cycle duration in an ovarian cancer cell is 36 hours [17], and is defined as the time between cell divisions in the absence of cell death. This implies that the division rate is: $b = \frac{1}{36}[\frac{1}{hour}] = 0.667[\frac{1}{day}]$. Late–stage HGSOC tumors double in volume approximately every 2.5 months [1], leading to net growth rate $\lambda = \frac{log(2)}{DT} = 0.0058[\frac{1}{day}]$, where DT is the doubling time. To achieve the observed λ, we set the cell death rate to $d = \lambda - b$. In our simulations, the parameters b and d are the same for all cell types and constant in absence of treatment.

Size of tumors at clinical diagnosis and chemotherapy effect

We modeled the probability of diagnosis previously as a function of tumor size as follows [11]. First, we estimated tumor burden using metabolic tumor volume (MTV) values extracted from ^{18}F-FDG-PET/CT images. Next, the MTV values were converted to a number of cells assuming 10^9 cells in 1cm^3 tumor bulk according to [3]. The number of cells was fitted to normal, exponential, Weibull, log–normal, logistic, and log–logistic probability distributions. The Bayesian Information Criterion (BIC) was calculated to measure the goodness of fit (GoF). The best agreement with the data was obtained for log–normal probability distribution with parameters $\mu = 26.59$ and $\sigma = 0.47$. Converting μ of the log–normal distribution to μ of the normal distribution, the total number of cancer cell at diagnosis equals $M = 3.9548 \cdot 10^{11}$ cells.

Patients in the calibration cohort received a median of three cycles of NACT where each cycle duration is 21 days. This means $\tau_{NACT} = 63$ [days] of NACT. Without loss of generality, we can assume that τ_{NACT} is also the time interval between the acquisition of PET/CT images before and after NACT. Parameter $d_{chemotherapy}$ was thus estimated for each patient separately as follows. We set all parameters except $d_{chemotherapy}$ and M to default values listed in Table 2. Next, for each patient, we extracted a number of cells before (M) and after (M_{NACT}) chemotherapy. In addition, we know that time between two PET/CT scans is $\tau_{NACT} = 3$ [weeks]. Thus, we estimated $d_{chemotherapy}$ numerically by with the bisection method to find a minimum of the following objective function:

$$J = M_{\hat{NACT}} - M_{NACT} = \sum_{i=1}^{n} x_i(t_{NACT}) - M_{NACT},$$

where $\sum_{i=1}^{n} x_i(t_{NACT})$ is total number of cancer cells from the model simulations after NACT.

Debulking surgery effect

The optimal debulking surgery is defined as a residual disease of 10 [mm] or fewer [18]. Tumor with 10 mm diameter corresponds to tumor volume of 10 [cm^3]. HGSOC is diagnosed when the tumor burden is of order 10^{11} cells. Thus, optimal surgery removes at the minimum fraction of $\beta \frac{10^9}{10^{11}}$ and corresponds to two cell–log kill fraction of cells removed by surgery.

Rate of resistance accumulation

We utilized clinical data from Kozłowska et al. 2018 to estimate the value of u [11]. Using a cohort of 33 HGSOC patients (training cohort), we performed Kaplan–Meier analysis to create platinum–free interval survival plot from the patient data.

Next, for a wide range of transition rate (u), the weight of chemotherapy effect on cells with one platinum resistance mechanism accumulated (α_1), and weight of chemotherapy effect on cells with two platinum resistance mechanisms (α_2), we performed a grid search. We took 100 values of u between $10^{-8} - 10^{-4}$ and 101 values of α_1 and α_2 between $0 - 1$ leading to 1,020,100 parameter combinations and performed simulations of 1,000 HGSOC patients for each parameter combination.

For each simulated cohort, Kaplan–Meier analysis was performed, and platinum-free interval survival plot was created. Next, the obtained survival plot from simulations was compared with one from the training cohort. Mean squared error (MSE) was calculated to measure the deviation between the two survival estimates. To take into account stochasticity of model simulations, for each value of u, 100 virtual HGSOC patient cohorts were simulated, and the mean value of MSE was calculated as a GoF estimate. The smallest MSE was obtained for: $u = 10^{-5}$, $\alpha_1 = 0.02$ and $\alpha_2 = 0.01$, and was set as a nominal value in our simulations.

Targeted treatment parameters

The model includes three targeted treatment drugs: trientine, Wee1 inhibitor, and birinapant. The drugs are incorporated in the model of platinum drug resistance in HGSOC by changing corresponding model parameter value (see Supplementary Text 1), *i.e.*, α_1 and α_2 (trientine), u_{ddr} (Wee1 inhibitor) and d_a (birinapant).

To estimate values of the treatment parameters, first, we estimated total relative drug efficacy using efficacy values from: *in vitro*, *in vivo* and early clinical trials, which were extracted from the literature. The Table S1 lists relative drug efficacy at three levels of evidence: *in vitro* (I), *in vivo* (V) and early clinical (C), which were applied to calculate total relative drug efficacy (E) using the following formula:

$$E = 0.2 \cdot I + 0.3 \cdot V + 0.5 \cdot C.$$

**drug**	***in vitro* (I)**	***in vivo* (V**	**early clinical (C)**	**total (E)**
Trientine	50 % [14]	75 % [2]	16 % [4]	41 %
Wee1 inhibitor	160 % [8, 16]	50% [19]	76% [13]	85 %
Birinapant	57% [20]	50% [9]	18% [15]	35%

Table S1: Estimated relative drug efficacy for: trientine, Wee1 inhibitor, and birinapant.

The values of E were applied to fit treatment parameters for selected drugs as follows. First, for each drug, ten parameter values (p_i) were taken uniformly between $[p_{min}, p_{max}]$. Next, for each value of p, the total relative efficacy was estimated via simulations of virtual HGSOC patients. The value of p is selected in such a way that E from the model simulations agree with the E extracted from literature (see Table S1).

Estimation of E from the model simulation requires further comment. We assume that the total relative drug efficacy could be measured by the tumor burden (the total number of cancer cells) after the first-line treatment, and estimated with the following formula:

$$\hat{E} = \frac{\hat{X}_i}{\hat{X}_j},$$

where X_i is a sum of all cancer cells from the model simulations after first-line treatment (*i.e.*, SOC alone), and X_j is the sum of all cancer cells from the model simulations after first-line treatment (*i.e.*, SOC and targeted therapy). Thus, the expected tumor burden after the treatment with SOC and targeted therapy equals:

$$\hat{X}_i = \hat{E} \cdot \hat{X}_j,$$

and the goal of targeted treatment parameter calibration is to find the parameter value where $\hat{E}$ from model simulation equals the one estimated and listed in Table S1 (E).

For a given drug, the following procedure was performed to estimate tumor burden after primary treatment as a function of p. For each of ten values of p, a cohort of 1,000 HGSOC virtual patients was simulated. As the model stochastic and thus, it is computationally intensive to simulate a large cohort of HGSOC virtual patients, we performed bootstrapping to estimate the value of p for each drug together with confidence intervals. Thus, from a cohort of 1,000 HGSOC virtual patients, 1,000 patients were sampled with replacement, and average tumor burden after primary treatment ($\hat{X}_j$) was extracted. The procedure of sampling and extracting $\hat{X}_j$ is performed for all ten values of p, and plot of $\hat{X}_j$ as a function of p was created. Next, linear regression for performed to estimate coefficients of a linear function. As the last step, the value of p for a given drug was estimated using the formula:

$$\hat{p}_i = \frac{\hat{X}_i - \alpha_1}{\alpha_2},$$

where α_1 and α_2 are coefficients of linear function.

The procedure from creation of bootstrap sample to estimation of p using above equation was performed 1,000 times. The value of p for a given drug is then calculated as a mean value over 1,000 estimates and 95% confidence interval around estimated p_{drug} was calculated. The pseudocode describing the method to estimate targeted treatment parameters is presented in Algorithm 1 and the Table S2 presents estimated targeted treatment parameters together with confidence intervals.

Algorithm 1 Estimation of targeted treatment parameters

procedure Fit targeted treatment parameter
  Calculate: $X_i = \hat{E} \cdot \hat{X}_j$
  Set: p_{min} , p_{max} and $N_{patients}$
  Simulate HGSOC virtual patient cohort for each p_j**:**
  for p_j = linspace(p_{min}, p_{max}, 20) **do**
    SimulateVirtualPatientsCohort(p_j)
  end for
  Set: $N \Leftarrow 1000$
  Estimate: p
  for i = 1 to N **do**
    for p_j = linspace(p_{min},p_{max}, 20) **do**
      Sample 1,000 HGSOC virtual patients with replacement simulated for $p = p_j$
      Calculate: $\hat{X}_i = \frac{\sum TumorBurdenAfterTreatment_j}{N_{patients}}$
    end for
    Plot X_i as a function of p
    Perform linear regression to estimate α_1 and α_2 of: $X_i = \alpha_1 \cdot P_j + \alpha_2$
    Calculate: $\hat{p}_i = \frac{X_i - \alpha_1}{\alpha_2}$

  end for
  Calculate: $p_{drug} = \frac{\sum p_i}{N}$ and confidence intervals
end procedure

**Drug**	**Parameter name**	**Parameter value**	**Confidence intervals**
trientine	α_1, α_2	$\alpha_1 = 0.148, \alpha_2 = 0.138$	$\alpha_1 = 0.136 - 0.161, \alpha_2 = 0.126 - 0.151$
wee1 inhibitor	u_{ddr}	0.069	0.065-0-075
birinapant	d_a	0.069	0.06-0-079

Table S2: Parameter values of targeted treatment parameters.

References

[1] Patrick O Brown and Chana Palmer. The preclinical natural history of serous ovarian cancer: Defining the target for early detection. *PLoS Medicine*, 6(7):e1000114, jul 2009.

[2] Szu-Jung Chen, Ching-Chuan Kuo, Hsin-Yi Pan, Tsui-Chun Tsou, Szu-Ching Yeh, and Jang-Yang Chang. Mechanistic basis of a combination d-penicillamine and platinum drugs synergistically inhibits tumor growth in oxaliplatin-resistant human cervical cancer cells in vitro and in vivo. *Biochemical Pharmacology*, 95(1):28–37, may 2015.

[3] Ugo Del Monte. Does the cell number 109 still really fit one gram of tumor tissue? *Cell Cycle*, 8(3):505–506, 2009.

[4] Siqing Fu, Ming Mo Hou, Jennifer Wheler, David Hong, Aung Naing, Apostolia Tsimberidou, Filip Janku, Ralph Zinner, Sarina Piha-Paul, Gerald Falchook, Macus Tien Kuo, and Razelle Kurzrock. Exploratory study of carboplatin plus the copper-lowering agent trientine in patients with advanced malignancies. *Investigational New Drugs*, 32(3):465–472, jun 2014.

[5] Siqing Fu, Aung Naing, Caroline Fu, Macus Tien Kuo, and Razelle Kurzrock. Overcoming platinum resistance through the use of a copper-lowering agent. *Molecular cancer therapeutics*, 11(6):1221–5, jun 2012.

[6] L Galluzzi, L Senovilla, I Vitale, J Michels, I Martins, O Kepp, M Castedo, and G Kroemer. Molecular mechanisms of cisplatin resistance. *Oncogene*, 31(15):1869–1883, 2012.

[7] Daniel T. Gillespie. Exact stochastic simulation of coupled chemical reactions. *The Journal of Physical Chemistry*, 81(25):2340–2361, dec 1977.

[8] H. Hirai, Y. Iwasawa, M. Okada, T. Arai, T. Nishibata, M. Kobayashi, T. Kimura, N. Kaneko, J. Ohtani, K. Yamanaka, H. Itadani, I. Takahashi-Suzuki, K. Fukasawa, H. Oki, T. Nambu, J. Jiang, T. Sakai, H. Arakawa, T. Sakamoto, T. Sagara, T. Yoshizumi, S. Mizuarai, and H. Kotani. Small-molecule inhibition of Wee1 kinase by MK-1775 selectively sensitizes p53-deficient tumor cells to DNA-damaging agents. *Molecular Cancer Therapeutics*, 8(11):2992–3000, nov 2009.

[9] D M Janzen, E Tiourin, J a Salehi, D Y Paik, J Lu, M Pellegrini, and S Memarzadeh. An apoptosis-enhancing drug overcomes platinum resistance in a tumour-initiating subpopulation of ovarian cancer. *Nature communications*, 6:7956, 2015.

[10] Deepak Kilari, Kenneth A Iczkowski, Chintan Pandya, Adam J Robin, Edward M Messing, Elizabeth Guancial, and Eric S Kim. Copper Transporter-CTR1 Expression and Pathological Outcomes in Platinum-treated Muscle-invasive Bladder Cancer Patients. *Anticancer research*, 36(2):495–501, feb 2016.

[11] Emilia Kozłowska, Anniina Färkkilä, Tuulia Vallius, Olli Carpén, Jukka Kemppainen, Seija Grénman, Rainer Lehtonen, Johanna Hynninen, Sakari Hietanen, and Sampsa Hautaniemi. Mathematical Modeling Predicts Response to Chemotherapy and Drug Combinations in Ovarian Cancer. *Cancer Research*, 78(14):4036–4044, jul 2018.

[12] Suzanne Leijen, Jos H Beijnen, and Jan H M Schellens. Abrogation of the G2 checkpoint by inhibition of Wee-1 kinase results in sensitization of p53-deficient tumor cells to DNA-damaging agents. *Current clinical pharmacology*, 5(3):186–91, aug 2010.

[13] Suzanne Leijen, Robin M.J.M. Van Geel, Gabe S. Sonke, Daphne De Jong, Efraim H. Rosenberg, Serena Marchetti, Dick Pluim, Erik Van Werkhoven, Shelonitda Rose, Mark A. Lee, Tomoko Freshwater, Jos H. Beijnen, and Jan H.M. Schellens. Phase II study of WEE1 inhibitor AZD1775 plus carboplatin in patientswith tp53-mutated ovarian cancer refractory or resistant to first-line therapy within 3 months. *Journal of Clinical Oncology*, 34(36):4354–4361, dec 2016.

[14] Zheng D. Liang, Yan Long, Wen Bin Tsai, Siqing Fu, Razelle Kurzrock, Mihai Gagea-Iurascu, Fan Zhang, Helen H.W. Chen, Bryan T. Hennessy, Gordon B. Mills, Niramol Savaraj, and Macus Tien Kuo. Mechanistic basis for overcoming platinum resistance using copper chelating agents. *Molecular Cancer Therapeutics*, 11(11):2483–2494, nov 2012.

[15] Anne M Noonan, Kristen P Bunch, Jin Qiu Chen, Michelle A Herrmann, Jung Min Lee, Elise C Kohn, Ciara C O'Sullivan, Elizabeth Jordan, Nicole Houston, Naoko Takebe, Robert J Kinders, Liang Cao, Cody J Peer, W Douglas Figg, and Christina M Annunziata. Pharmacodynamic markers and clinical results from the phase 2 study of the SMAC mimetic birinapant in women with relapsed platinum-resistant or -refractory epithelial ovarian cancer. *Cancer*, 122(4):588–597, feb 2016.

[16] Abdullah A Osman, Marcus M Monroe, Marcus V Ortega Alves, Ameeta A Patel, Panagiotis Katsonis, Alison L Fitzgerald, David M Neskey, Mitchell J Frederick, Sang Hyeok Woo, Carlos Caulin, Teng-Kuei Hsu, Thomas O McDonald, Marek Kimmel, Raymond E Meyn, Olivier Lichtarge, and Jeffrey N Myers. Wee-1 kinase inhibition overcomes cisplatin resistance associated with high-risk TP53 mutations in head and neck cancer through mitotic arrest followed by senescence. *Molecular cancer therapeutics*, 14(2):608–19, feb 2015.

[17] John Carl Panetta. A mathematical model of breast and ovarian cancer treated with paclitaxel. *Mathematical Biosciences*, 146(2):89–113, dec 1997.

[18] John O Schorge, Christopher McCann, and Marcela G Del Carmen. Surgical debulking of ovarian cancer: what difference does it make? *Reviews in obstetrics & gynecology*, 3(3):111–7, 2010.

[19] A. A. Van Linden, D. Baturin, J. B. Ford, S. P. Fosmire, L. Gardner, C. Korch, P. Reigan, and C. C. Porter. Inhibition of Wee1 Sensitizes Cancer Cells to Antimetabolite Chemotherapeutics In Vitro and In Vivo, Independent of p53 Functionality. *Molecular Cancer Therapeutics*, 12(12):2675–2684, dec 2013.

[20] K P Zeligs, F Ning, L Hernandez, T Smyth, G Ward, and C M Annunziata. IAP antagonism enhances anti-tumor activity of docetaxel or panobinostat in ovarian cancer cell lines. *Gynecologic Oncology*, 149:13, jun 2018.

Supplementary figure 1

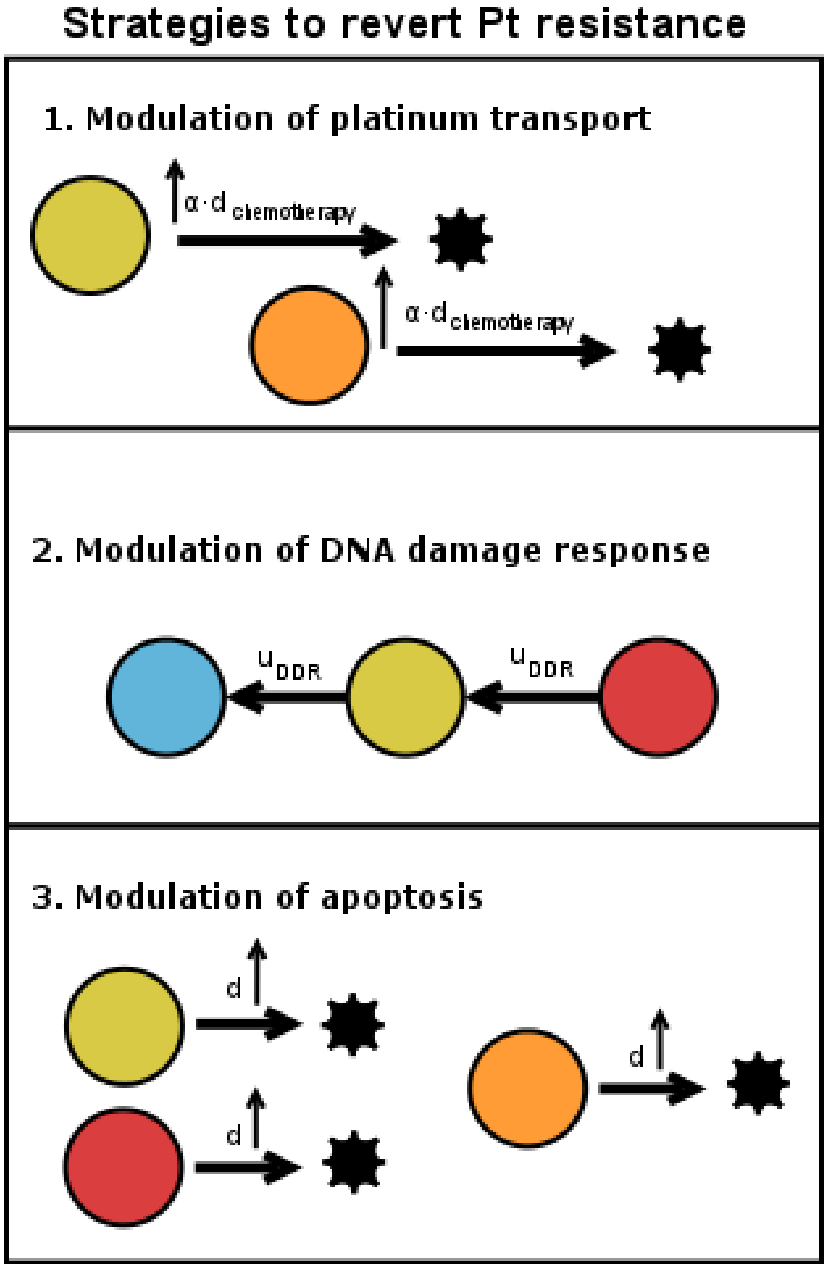

Figure S1: **Schematic shows inclusion of targeted therapy into the model of innate platinum resistance.** Three types of drugs which target a given platinum resistance mechanism are included in the model: trientine, Wee1 inhibitor and birinapant.

Supplementary figure 2

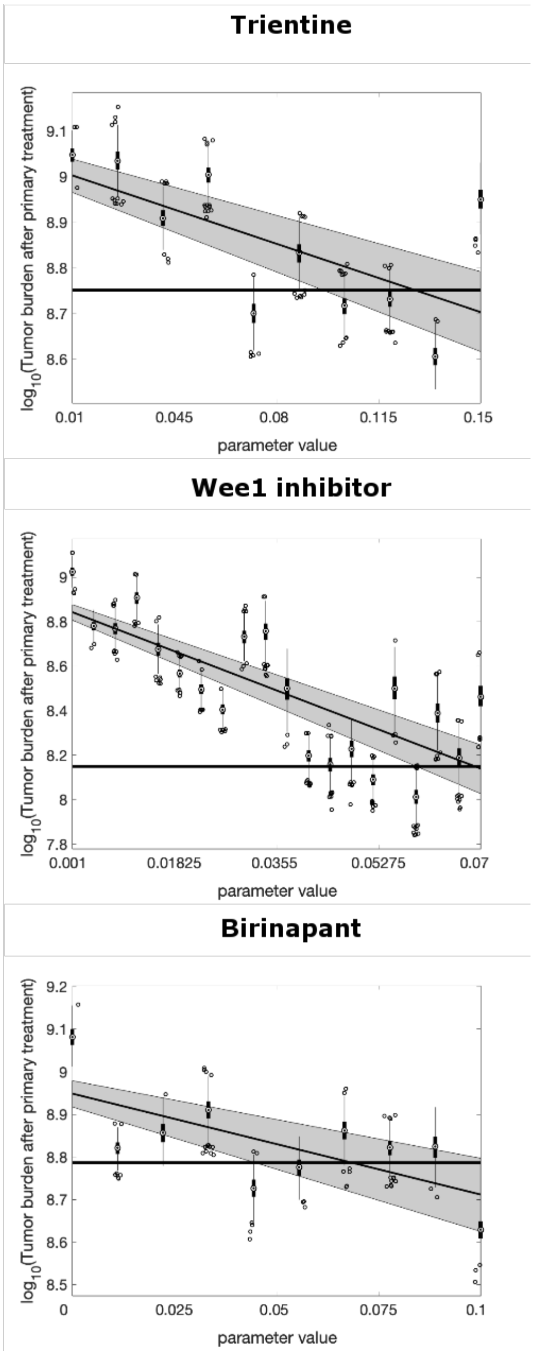

Figure S2: **Calibration of targeted treatment parameters** The figures show tumor burden after primary treatment as a function of p. The horizontal line shows the expected tumor burden after primary treatment and the black line show the model fit. The gray area represents confidence intervals and the boxplots the estimated tumor burden after primary treatment for a given value of p.

Supplementary figure 3

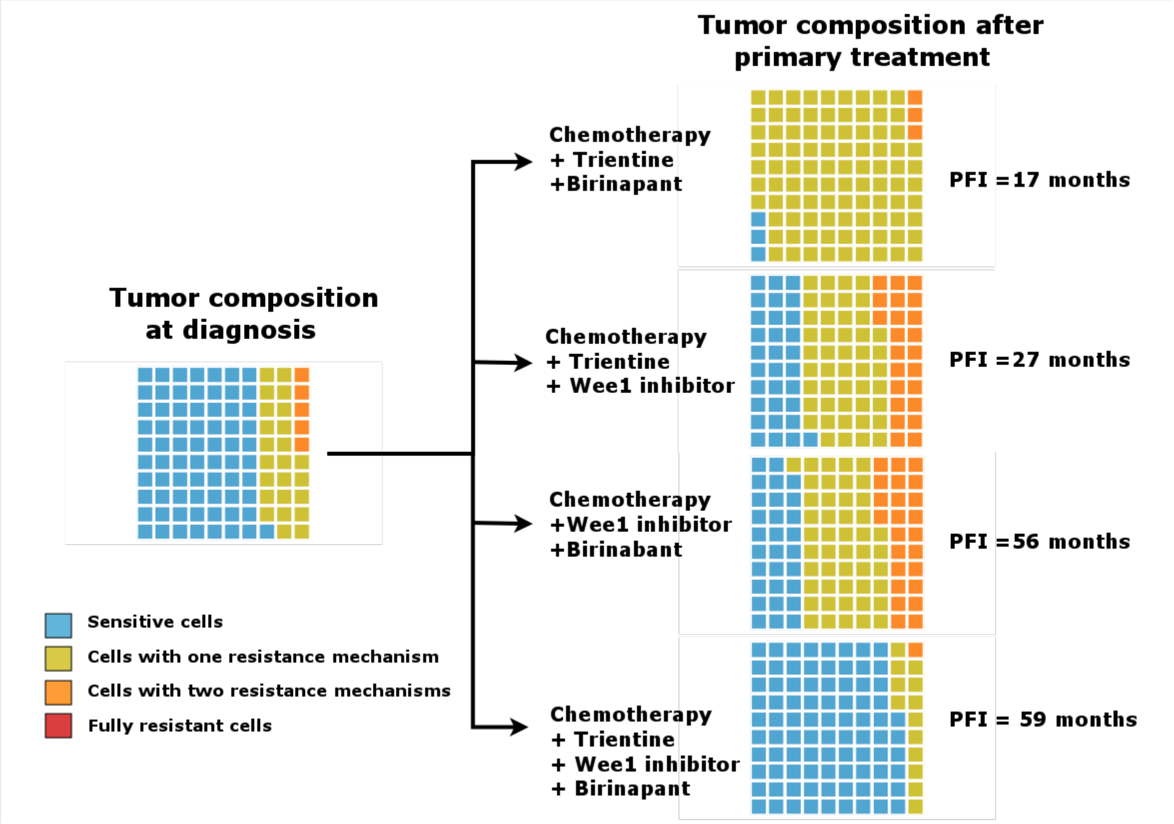

Figure S3: **Result from virtual clinical trial simulations of two and three drugs combinations.** The waffle plots illustrate tumor composition after primary treatment. Blue, yellow, orange and red color represent sensitive, cells with one platinum resistance mechanism active, cells with two platinum resistance mechanisms active and fully resistant cells.
